# Sustainable hemocompatibility prediction of electrospun nanomaterials using SEM-driven CNN ensemble learning aligned with ISO 10993

**DOI:** 10.1371/journal.pone.0359594

**Published:** 2026-10-05

**Authors:** Narasimhan Kumaravelu, Adalarasu Kanagasabai, Preetham Raj Aravindan, Surya Murugan Moorthy Sathyanarayana, Saravana Kumar Jaganathan

**Affiliations:** 1 School of Electrical and Electronics Engineering, SASTRA Deemed to be University, Thanjavur, Tamil Nadu, India; 2 Institute of Research and Development, Duy Tan University, Da Nang, Vietnam; 3 School of Engineering & Technology, Duy Tan University, Da Nang, Vietnam; 4 Biomedical Engineering Research Group, School of Engineering, University of Leicester, Leicester, United Kingdom; Université de Rouen Normandie, FRANCE

## Abstract

**Background:**

Electrospun nanocomposite biomaterials are promising candidates for blood-contacting medical devices owing to their high surface area, tunable nanoscale morphology, and biomimetic replication of the native extracellular matrix. However, conventional hemocompatibility assessments rely heavily on biological assays. These methods require blood samples, specialized reagents, and consumables, thereby inherently increasing costs, resource demands, and laboratory waste. To mitigate these limitations, sustainable evaluation strategies that leverage physicochemical features are highly desirable.

**Methodology:**

In this study, we present a deep learning framework for predicting the hemocompatibility of electrospun nanofibres directly from scanning electron microscopy (SEM) images. Pretrained convolutional neural network models, namely VGG19, ResNet50, and InceptionV3, were fine-tuned to classify scanning electron microscopy (SEM) images of nanofibres as haemocompatible or non-haemocompatible. To ensure data robustness, an Albumentations-based augmentation strategy was implemented, and predictive reliability was further optimized using a majority-voting ensemble learning approach.

**Results:**

Among the individual architectures evaluated, InceptionV3 demonstrated superior performance, achieving a precision of 0.98, a recall of 1.00, and an F1-score of 0.99. The ensemble model exhibited comparable robustness, reaching an overall accuracy of 99%. Furthermore, explainable AI (XAI) techniques, specifically LIME and Integrated Gradients, confirmed that the network’s predictions were driven by physiologically relevant microstructural features of the nanofibres.

**Conclusion:**

This artificial intelligence-assisted, SEM image-based physicochemical approach offers an eco-friendly pre-screening strategy that significantly reduces reliance on blood-based assays and associated laboratory consumables. Ultimately, this framework aligns with ISO 10993 guidelines by using SEM-derived physicochemical information as a pre-screening tool to support subsequent biological evaluation, thereby enabling efficient, regulatory-compliant biomaterial development.

## 1. Introduction

Biomaterials play a central role in modern medicine by enabling the repair, replacement, and regeneration of damaged tissues, thereby restoring physiological function and improving patient outcomes. The global biomaterials market, valued at approximately USD 178 billion in 2023, is projected to reach nearly USD 489 billion by 2030, reflecting rapid clinical and commercial expansion [1]. These materials are widely used in implants, drug delivery systems, tissue scaffolds, and cardiovascular devices.

Among fabrication techniques, electrospinning has emerged as a versatile and widely adopted method for producing fibrous biomaterials. It enables the formation of continuous micro- and nanofibres from polymer solutions or melts under a high-voltage electric field, resulting in structures with high surface area, tunable porosity, and extracellular matrix (ECM)-like architecture. These features make electrospun scaffolds highly suitable for tissue engineering, wound healing, and regenerative medicine applications, including vascular grafts, wound dressings, and cardiac patches [2]. Eelctrospun scaffolds can be engineered from biodegradable polymers and tailored into aligned or random structures to meet specific tissue requirements. Within the range of polymers commonly employed for electrospinning, polyurethane (PU) has attracted considerable attention due to its excellent elasticity, mechanical durability, and electrospinnability, enabling the fabrication of stable nanofibrous architectures capable of withstanding dynamic physiological conditions. These properties make PU particularly suitable for applications such as vascular grafts and cardiac patches, where mechanical compliance and long-term structural integrity are essential. However, despite these advantages, PU is intrinsically bioinert, which limits cell–material interactions and reduces its bio-functionality in blood-contacting environments [3].

To overcome this limitation, bioactive natural extracts have been incorporated into polymer matrices to introduce biochemical cues that enhance cell adhesion, proliferation, antimicrobial activity, and tissue regeneration responses. These bio-derived modifiers effectively convert inert scaffolds into bio-interactive interfaces. In parallel, inorganic nanoparticles such as bioactive ceramics and metal oxides have been widely explored to reinforce mechanical stability and regulate interfacial biological responses, including hemocompatibility and antimicrobial behaviour [4]. The integration of polymeric matrices with natural bioactive and inorganic phases therefore enables the development of multifunctional scaffolds with synergistically enhanced structural and biological performance [5]. Such hybrid systems are particularly relevant for blood-contacting applications, including vascular grafts and cardiac patches, where simultaneous requirements of mechanical resilience, hemocompatibility, and bioactivity must be satisfied.

Despite these advantages, haemocompatibility evaluation remains a major bottleneck in biomaterials development. According to ISO 10993 standards [6], haemocompatibility testing is required as part of the biological evaluation of medical devices; however, conventional assays such as platelet adhesion, haemolysis, and coagulation tests are time-consuming, costly, and resource intensive. These methods require human blood samples, specialised reagents, and single-use consumables, raising ethical, financial, and environmental concerns. Collectively, these limitations highlight the need for faster, more sustainable, and predictive pre-screening approaches.

In this context, artificial intelligence provides a natural extension of existing characterisation workflows by enabling data-driven analysis of routinely acquired material data. In particular, deep learning models can extract meaningful patterns from scanning electron microscopy (SEM) images of biomaterial surfaces to identify morphology-associated haemocompatibility trends prior to extensive experimental validation. As a pre-screening strategy, this approach reduces reliance on early-stage wet-lab assays, thereby decreasing cost, resource consumption, and experimental waste while accelerating material screening workflows. Importantly, the proposed framework is intended to support, rather than replace, conventional haemocompatibility testing required under ISO 10993 standards.

Recent advances in deep learning, particularly convolutional neural networks (CNNs) combined with transfer learning, have shown strong performance in biomedical image classification tasks. In this study, transfer learning is applied using three established CNN architectures, namely VGG19, ResNet50, and InceptionV3, to classify SEM images of electrospun nanofibres. These models are integrated using an ensemble majority voting strategy to improve robustness and reduce model-specific bias [7,8]. However, the use of deep learning in biomedical applications is often limited by a lack of interpretability, as models are frequently considered black-box systems. To address this limitation, explainable artificial intelligence (XAI) techniques, including Local Interpretable Model-Agnostic Explanations (LIME) and Integrated Gradients, are incorporated to provide transparent and interpretable insights into model decision-making by highlighting the image regions influencing classification outcomes [9].

To the best of our knowledge (Fig 1), this study represents the first systematic investigation of haemocompatibility classification of electrospun biomaterials directly from SEM images using deep learning combined with explainable AI. The novelty of this work lies in integrating SEM-based surface morphology analysis with ensemble deep learning and XAI to enable predictive hemocompatibility screening. Unlike existing studies that rely solely on experimental assays or conventional material characterisation, this approach introduces a non-destructive, image-driven predictive framework for early-stage biomaterial evaluation.

**Fig 1:**
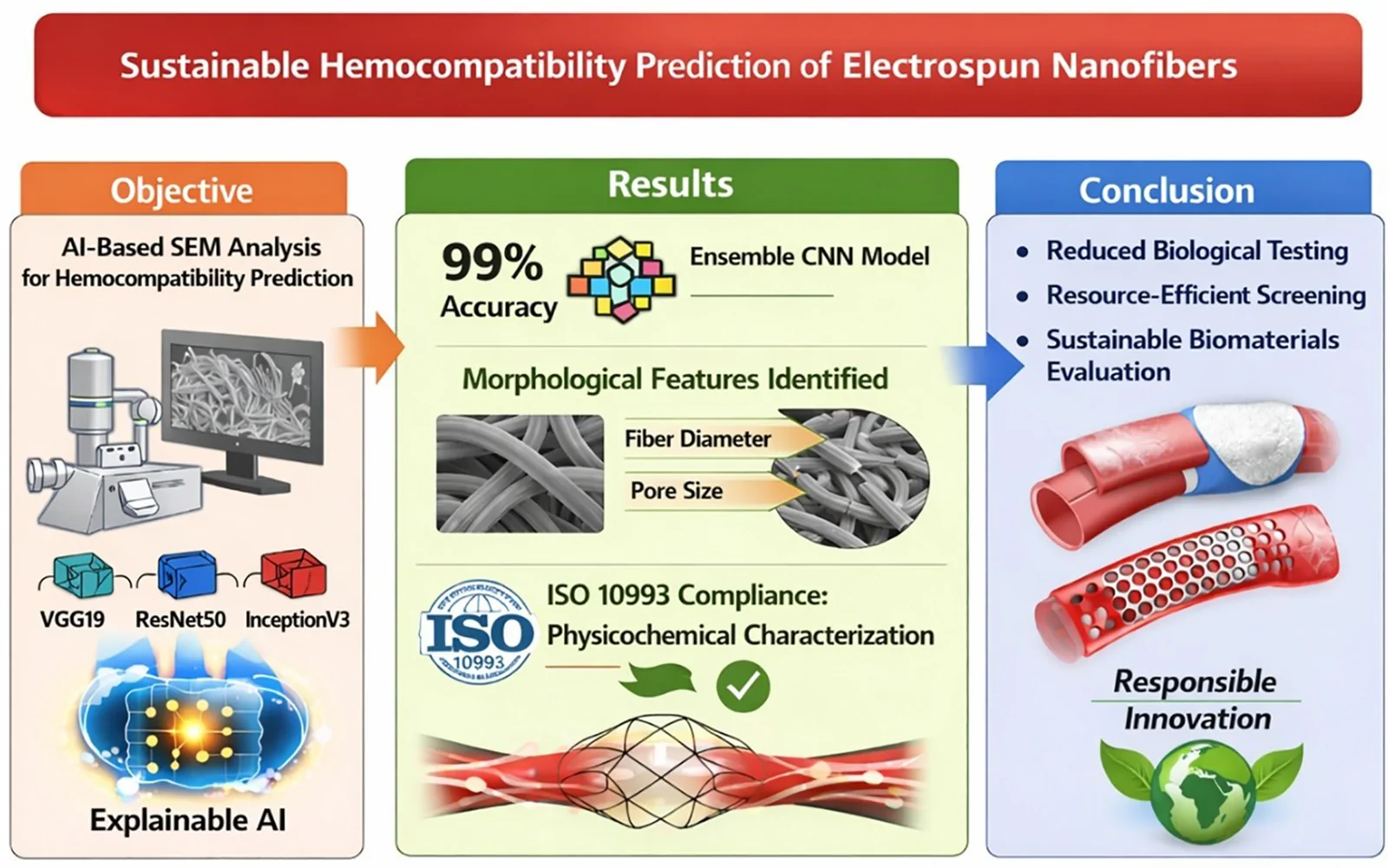
Graphical abstract illustrating a sustainable framework for hemocompatibility prediction of electrospun nanofibres. SEM images of nanofibres are analyzed using an ensemble of pretrained convolutional neural networks—VGG19, ResNet50, and InceptionV3—integrated with Explainable AI (LIME and Integrated Gradients) to identify key morphological features such as fiber diameter, pore size, and orientation. The workflow highlights SEM-based physicochemical analysis as a sustainable pre-screening tool, supporting reduction of biological assays and alignment with ISO 10993. The figure was generated with assistance from ChatGPT.

The proposed method provides a sustainable and resource-efficient pre-screening tool that aligns with ISO 10993 principles while supporting, not replacing, final regulatory biological testing. This framework bridges artificial intelligence and biomaterials science, enabling faster screening, improved reproducibility, and enhanced translational potential for blood-contacting medical devices.

The key contributions of this study are as follows. First, a deep learning framework is developed for binary classification of haemocompatible and non-haemocompatible electrospun nanofibres using SEM images. Second, Albumentations-based data augmentation and preprocessing strategies are applied to improve generalisation and address data imbalance. Third, an ensemble voting classifier integrating VGG19, ResNet50, and InceptionV3 is employed to reduce model-specific bias. Fourth, explainable AI techniques, including LIME and Integrated Gradients combined with superpixel segmentation, are used to enhance interpretability and user trust. Finally, the framework is evaluated using standard performance metrics, ROC analysis, and ablation studies.

The remainder of this paper is organised as follows. Section 2 reviews prior work on Electrospun materials, hemocompatibility assessment, image-based biomaterial analysis, ensemble learning, and explainable AI. Section 3 describes the dataset, preprocessing, deep learning models, ensemble strategy, and XAI framework. Section 4 presents experimental results. Section 5 discusses implications, limitations, and real-world applicability, followed by conclusions.

## 2. Related works

Polyurethane (PU) has been widely investigated as a base polymer for electrospun nanofibrous scaffolds in cardiovascular and soft tissue engineering due to its exceptional elasticity, fatigue resistance, and mechanical stability under cyclic deformation. These properties closely resemble native vascular tissue mechanics, making PU particularly suitable for vascular grafts and cardiac patch applications. Electrospinning as a fabrication technique enables the production of nanofibrous structures with extracellular matrix (ECM)-like architecture and high surface area, which has been extensively reviewed for biomedical applications [10]. However, pristine PU exhibits poor bioactivity and limited hemocompatibility due to its hydrophobic surface chemistry and absence of specific cell-binding motifs, which restrict endothelial cell adhesion and promote non-favourable protein adsorption profiles [11]. Consequently, PU is increasingly regarded as a structural framework material that requires functional enhancement for biomedical performance in blood-contacting environments. In this context, Jaganathan and Mani (2018) demonstrated that polyurethane-based electrospun nanofibres can be effectively engineered for vascular applications by incorporating bioactive modifications to improve hemocompatibility and reduce thrombogenic responses [4]. Their findings highlight that surface modification and composite design significantly enhance biological interactions while maintaining the mechanical integrity of PU scaffolds, reinforcing its suitability as a tunable base material for cardiovascular tissue engineering.

To overcome the intrinsic bio-inertness of polymeric scaffolds, bioactive natural extracts have been extensively incorporated into electrospun systems to introduce biochemical signalling capability. Plant-derived compounds such as polyphenols, flavonoids, and essential oils have been reported to enhance antimicrobial activity, reduce oxidative stress, and promote fibroblast adhesion and proliferation [12]. These bioactive agents effectively convert inert polymeric matrices into biointeractive systems capable of supporting wound healing and tissue regeneration. However, a major limitation is their rapid diffusion and burst release behaviour, which can reduce long-term stability and sustained bioactivity within the scaffold environment. To resolve these limitations and form a truly synergistic architecture, multicomponent scaffolding platforms such as those blending polyurethane matrices with essential oils and natural products have been engineered to achieve a more favorable, hydrophilicity-balanced, and controlled cellular environment [5]. In parallel, inorganic nanoparticles such as hydroxyapatite, zinc oxide (ZnO), titanium dioxide (TiO_2_), and other metal oxides have been integrated into polymeric nanofibres to enhance mechanical reinforcement and introduce additional functional bioactivity. Electrospun polymer–inorganic composite nanofibres have been widely studied for biomedical applications due to their improved structural stability and tunable surface properties. These nanoparticles improve scaffold stiffness, surface wettability, and blood compatibility. In PU-based composite systems, nanoparticle incorporation has been shown to significantly improve structural integrity and hemocompatibility compared to pristine polymer scaffolds, making them suitable for load bearing and blood-contacting biomedical applications [13].

Modern biomaterials research has evolved beyond the concept of bioinertness toward the design of biologically active materials that actively integrate with host tissues, promote healing, and elicit favorable biological responses rather than merely avoiding adverse effects [14]. Blood compatibility, or hemocompatibility, represents a regulatory and safety cornerstone for any material or device intended for blood contact. ISO 10993−4 defines the standard framework and recommended in vitro and in vivo tests for evaluating blood–material interactions, and the outcomes of these assessments inform subsequent animal and clinical studies. Standard in vitro assays include hemolysis testing to quantify erythrocyte lysis, platelet adhesion and activation assays, complement activation measurements, and coagulation tests such as activated partial thromboplastin time and prothrombin time, often conducted under static or controlled shear conditions to approximate physiological flow. While these biochemical and cellular assays provide mechanistic insight into blood–material interactions, they are time consuming, reagent intensive, and reliant on fresh blood, specialized facilities, and trained personnel. Consequently, their throughput is limited when screening large biomaterial libraries, motivating the development of rapid *in-silico* or image based prescreening strategies to prioritize candidates for experimental validation [15].

Digital microscopy and histopathology imaging have emerged as rich sources of phenotypic information for characterizing blood–material interactions, including surface deposition, thrombus formation, and cellular adhesion patterns. Over the past decade, convolutional neural networks have become the dominant paradigm for medical image analysis due to their ability to learn hierarchical and translation invariant features directly from pixel data without manual feature engineering. Comprehensive reviews document the adaptation of convolutional neural networks for classification, detection, and segmentation tasks across diverse medical imaging modalities. Transfer learning, in which models pretrained on large-scale image datasets such as ImageNet are fine-tuned for domain-specific tasks, has become a widely adopted strategy in scientific and biomedical image analysis, particularly when acquiring large annotated datasets is challenging [16]. By leveraging features learned from millions of natural images, pretrained networks can effectively extract relevant visual patterns from specialized datasets while reducing the need for extensive training data. For example, transfer learning-based architectures incorporating U-Net, ResNet, and EfficientNet have been successfully applied to defect detection and surface-quality classification in manufactured metal components [17]. Similarly, pretrained VGG16 and ResNet50 networks have been used to classify scanning electron microscopy (SEM) images of pharmaceutical excipient powders based on their morphological characteristics [18]. These studies demonstrate that transfer learning provides robust feature initialization, reduces training time, and frequently outperforms models trained from scratch when only limited domain-specific data are available.

Ensemble learning approaches commonly combine diverse pretrained backbones, as each architecture encodes distinct inductive biases related to filter size, network depth, and connectivity, resulting in complementary feature representations. VGG19 has been extensively applied in materials science and biomaterials imaging for the classification of scanning electron microscopy (SEM) images and microstructural textures. Its deep stack of small convolutional filters enables the learning of fine-grained morphological patterns, such as fiber alignment, porosity, and surface roughness, in electrospun scaffolds and polymer composites. ResNet50 has been employed to analyze surface defects, classify alloy microstructures, and assess cell–material interactions on scaffold surfaces, with its residual connections facilitating the extraction of deep hierarchical features. InceptionV3, owing to its multi-scale convolutional architecture, efficiently captures both global structural information and local textural features and has been applied to the microscopy-based analysis of tissue scaffolds, polymer blends, and nanocomposites [19].

Each backbone architecture exhibits inherent strengths and limitations. VGG networks are parameter-intensive but conceptually simple, ResNet architectures enable the effective optimization of deeper networks, and Inception architectures provide computational efficiency through multi-scale feature extraction. Combining these models within an ensemble exploits complementary error patterns and feature specializations, thereby improving robustness to dataset variability. Importantly, improvements in ensemble performance arise from architectural diversity, transfer learning strategies, and domain-specific fine-tuning rather than from architectural complexity alone [20].

Empirical studies in medical imaging consistently demonstrate that pretrained convolutional neural networks fine-tuned in a layer wise manner outperform models trained from scratch when labeled data are scarce. The optimal depth of fine tuning depends on dataset size and domain similarity. Domain shift caused by variations in imaging devices, sample preparation, or staining remains a major challenge, and practical mitigation strategies include normalization and augmentation techniques [21].

Majority voting ensembles that aggregate predictions from VGG19, ResNet50, and InceptionV3 have been shown to improve classification accuracy and robustness by reducing variance and compensating for model specific errors. Albashish et al. demonstrated the effectiveness of majority voting and product rule ensembles in colon histopathology classification, where ensemble models consistently outperformed individual networks across internal and external datasets [22]. Systematic reviews in medical image analysis further confirm that ensembles of heterogeneous convolutional neural networks generally improve generalization performance, particularly on external validation datasets.

Nevertheless, ensemble learning does not guarantee substantial performance gains in all scenarios. Studies indicate that ensemble improvements depend on dataset complexity, architectural diversity, and error decorrelation among base models [23]. When a single model is already highly optimized, ensemble gains may be marginal. In such cases, ensembles provide benefits in robustness, uncertainty moderation, and risk reduction rather than accuracy alone, albeit at the cost of increased computational complexity.

Model interpretability remains essential for clinical translation. Explainable artificial intelligence methods such as Local Interpretable Model Agnostic Explanations and Integrated Gradients are increasingly employed in medical imaging to provide local and visually intuitive explanations of deep learning predictions, particularly in binary classification tasks. LIME generates explanations through localized perturbations, while Integrated Gradients produces attribution maps that satisfy axiomatic properties such as sensitivity and implementation invariance, making the two approaches complementary for clinical adoption [24]. Recent reviews highlight the growing role of machine learning in predicting biomaterial properties, optimizing scaffold architectures, and accelerating the design of advanced biomaterials and biofabrication processes. As predictive models become increasingly sophisticated, explainable artificial intelligence techniques are gaining importance for improving model transparency and interpretability, thereby supporting more informed and reliable decision-making in biomaterials research and tissue engineering applications [25].

LIME has been applied to interpret deep neural networks in biomedical classification tasks, including breast tumour classification, by providing local feature importance explanations that improve the interpretability of model predictions [26]. Reviews of explainable artificial intelligence in biomaterials research identify LIME as a versatile method applicable to image, tabular, and spectral data commonly encountered in material characterization, enabling actionable insights for materials design [27]. Integrated Gradients was originally introduced as a principled attribution method for deep networks, offering reliable explanations through gradient integration from baseline inputs [28]. Subsequent surveys in biomedical image analysis highlight its utility in generating pixel level attribution maps that link neural network predictions to tissue and cellular morphology, thereby enhancing transparency and clinical trust.

Together, these advances support hybrid evaluation strategies that combine artificial intelligence driven prescreening with targeted biochemical testing. Such approaches enable rapid prioritization of candidate materials with potential thrombogenic risk for further experimental validation, reducing time and cost while maintaining regulatory rigor. Enhancing the transparency of artificial intelligence predictions through explainable methods such as LIME and Integrated Gradients further supports compliance, trust, and clinical relevance. A summary of relevant studies employing convolutional neural networks and explainable artificial intelligence methods is provided in the Table 1.

**Table 1:** Key literature on polyurethane biomaterials, hemocompatibility assessment, and artificial intelligence-driven image analysis.

Reference	Year	Dataset/ Domain	Methodology	Application/ Findings
[10]	2017	Electrospun nanofibrous scaffolds	Review of electrospinning technologies	Demonstrated the ability of electrospinning to produce ECM-like nanofibrous architectures suitable for tissue engineering and regenerative medicine.
**[13]**	2022	PU-based composite biomaterials	Polymer–nanoparticle composite scaffolds	Showed that incorporation of inorganic nanoparticles improves mechanical properties, wettability, structural stability, and hemocompatibility of polymeric scaffolds.
[15]	2018	Blood-contacting biomaterials	Conventional hemocompatibility assessment methods	Highlighted limitations of blood compatibility assays and emphasized the need for rapid image-based and computational prescreening approaches.
[16]	2016	Medical imaging	Transfer learning with pretrained CNNs	Demonstrated that fine-tuned pretrained CNNs outperform models trained from scratch when annotated datasets are limited.
[20]	2018	Machine learning and image analysis	Ensemble learning survey	Reported that combining diverse architectures improves robustness, generalization, and predictive performance through complementary feature representations.
[22]	2023	Colon histopathology images	Majority voting and product-rule CNN ensembles	Showed that ensemble models consistently outperformed individual CNN architectures across internal and external datasets.
[25]	2024	Biomaterials and biofabrication	Machine learning and AI review	Highlighted the growing role of AI in biomaterial property prediction, scaffold optimization, and accelerated biomaterials discovery.
[24,28]	2016–2017	Explainable AI	LIME and Integrated Gradients	Introduced widely adopted XAI methods for interpreting deep learning models and improving transparency and trust in biomedical applications.

## 3. Methodology

### 3.1. Electrospinning

Electrospun nanofibrous membranes analysed in this study were fabricated and characterized previously using a medical grade polyurethane, Tecoflex EG 80A, selected for its well-established biocompatibility, flexibility, and suitability for biomedical applications. The polymer was blended with selected natural bioactive extracts and inorganic additives to modulate fiber morphology and functional properties. Specifically, natural extracts derived from beetroot, clove leaf, and grapefruit, as well as inorganic additives including cerium oxide and magnesium chloride, were incorporated into the polymer matrix, as reported in earlier studies.

The bioactive extracts were prepared using standard aqueous or ethanolic extraction protocols, followed by filtration and solvent removal to obtain concentrated extracts. Inorganic additives were dissolved or uniformly dispersed in dimethylformamide (DMF) prior to blending to ensure homogeneous distribution within the polymer solution. Electrospinning was conducted using a 9% (w/v) polyurethane solution dissolved in DMF. For the modified scaffolds, the concentration of incorporated bioactive components ranged from 4% to 9% (w/v) depending on the formulation. The solution was delivered through a 21-gauge stainless steel needle at a controlled flow rate ranging from 0.2 to 0.5 mL/h. A constant applied voltage of 10.5 kV was used, and the nanofibres were collected at a fixed tip-to-collector distance of 20 cm. All electrospinning experiments were performed under controlled environmental conditions at a temperature of 22 ± 2 °C and a relative humidity of 45 ± 5% to ensure process stability and reproducibility. Electrospinning was performed under optimized processing conditions to produce uniform, bead-free nanofibres. The resulting membranes were vacuum dried to remove residual solvents.

The surface morphology of the electrospun nanofibrous scaffolds was characterised using a Hitachi High-Technologies TM3030 tabletop scanning electron microscope operated at an accelerating voltage of 15 kV. Prior to imaging, all samples were sputter-coated with a thin layer of gold to improve surface conductivity and minimise charging effects during electron beam exposure. To ensure robust, reproducible, and unbiased morphological assessment, SEM micrographs were systematically acquired at three standardised magnifications (1,000 × , 3,000 × , and 5,000×) from n = 3 scaffold replicas for each formulation. This multi-scale imaging strategy enabled comprehensive evaluation of fibre morphology, surface uniformity, and structural consistency across different scaffold compositions. Furthermore, no specific scaffold thickness threshold was required for the proposed analysis, provided that the samples possessed sufficient structural integrity for stable SEM imaging and generation of high-quality micrographs with clearly resolved fibrous features and minimal imaging artefacts.

Dataset labels were assigned based on quantitative hemocompatibility assessments performed directly on the electrospun scaffolds. Specifically, activated partial thromboplastin time (APTT), prothrombin time (PT), and hemolysis assays were conducted to evaluate blood compatibility. The biological assay results obtained for each scaffold were subsequently correlated with the corresponding scanning electron microscopy (SEM) images. Based on the ISO 10993 guidelines for blood-contacting biomaterials, samples demonstrating acceptable hemocompatibility responses were labelled as haemocompatible, whereas samples exhibiting adverse blood interaction responses beyond acceptable limits were classified as non-haemocompatible

### 3.2. Deep learning techniques

The proposed classification framework employs an ensemble of three convolutional neural networks to distinguish between blood compatible and non-compatible electrospun nanofibrous membranes based on scanning electron microscopy images. Fig 2 presents a schematic overview of the framework used for blood compatibility prediction, illustrating the flow of data from raw input images through feature extraction and final classification. The framework integrates three established convolutional neural network architectures, namely VGG19, ResNet50, and InceptionV3, which operate in parallel to leverage their complementary representational capabilities. The individual predictions generated by each network are subsequently combined using a voting-based ensemble strategy to enhance robustness and overall classification performance. The following subsections describe the dataset preparation, network architectures, training strategy, ensemble decision rule, and model explainability approach.

**Fig 2:**
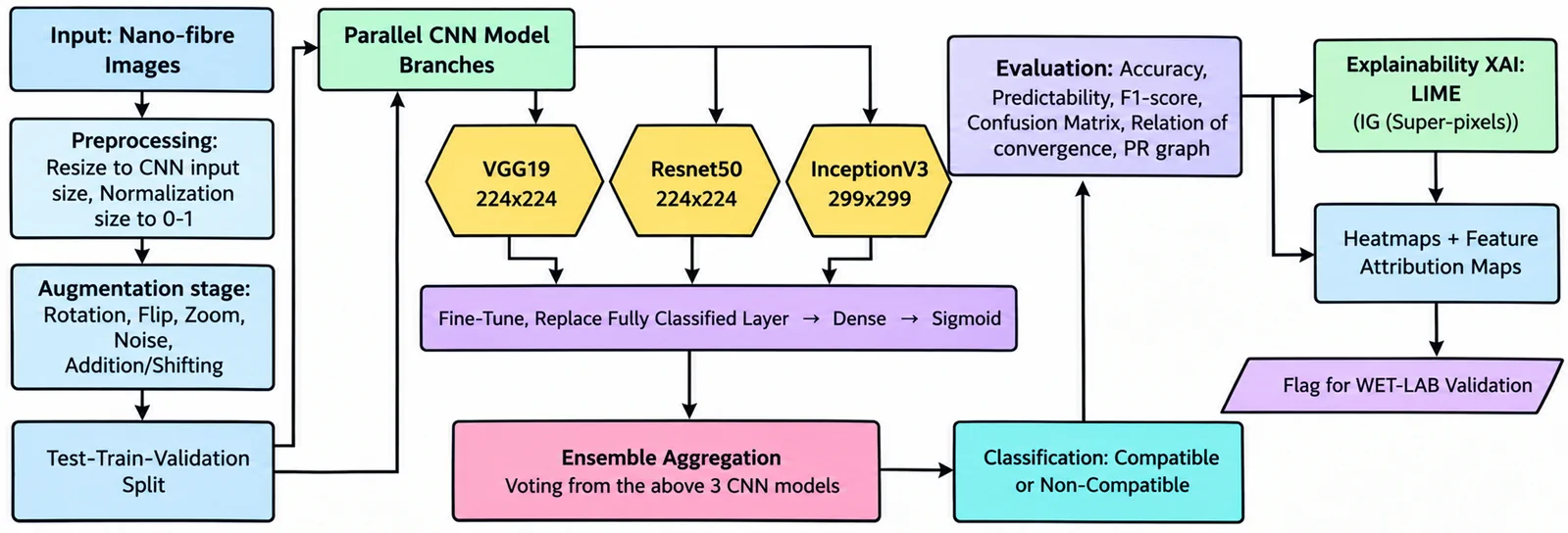
Flowchart of the proposed comprehensive experimental design.

For each backbone architecture, the original ImageNet classification head was replaced with a task specific binary classifier. This classifier comprises a global pooling layer followed by one or more fully connected layers with rectified linear unit activation and dropout regularization. Dropout is particularly important for medical imaging applications with limited datasets, as it reduces overfitting by randomly deactivating neurons during training and thereby improves model generalization. The final output layer consists of a single neuron with sigmoid activation to generate binary predictions.

The convolutional base of each network was initialized using ImageNet pretrained weights. During the initial training phase, the base layers were frozen, and only the newly added classification layers were trained on the electrospun fiber dataset. After convergence of the top layers, fine tuning was performed by selectively unfreezing deeper layers of the convolutional base. This two-stage training strategy allows the model to adapt high level feature representations to the specific characteristics of electrospun nanofibres while preserving robust low level feature extraction learned from large scale natural image data. Table 2 presents the complete set of training hyperparameters used for deep learning model development, encompassing optimizer settings, learning rate, batch size, number of epochs, dropout regularization, input image size, validation data allocation, and loss function. These parameters were selected to ensure stable convergence, effective feature learning, and robust model generalization.

**Table 2:** Model training hyperparameters for reproducible deep learning framework development.

Hyperparameter	Value
Optimizer	Adam
Learning rate	1 x (10^-4)
Batch size	4
Epochs	25
Dropout rate	0.6
Input Image size	224 x 224
Validation split	25%
Loss Function	Binary Cross Entropy

VGG19 is a nineteen-layer deep convolutional neural network composed of sixteen convolutional layers and three fully connected layers, characterised by a simple and uniform architecture based on small convolutional kernels. ResNet50 is a fifty-layer deep residual network that incorporates identity skip connections, enabling efficient training of deeper models by mitigating vanishing gradient issues. InceptionV3 is a deep inception architecture that employs parallel convolutional filters of varying sizes within inception modules, allowing efficient multi-scale feature extraction while maintaining computational efficiency. These architectures were selected for their complementary strengths and demonstrated effectiveness in biomedical image analysis, where VGG19 provides stable feature learning through architectural simplicity, ResNet50 ensures efficient optimization via residual learning, and InceptionV3 captures hierarchical spatial features across multiple scales with reduced computational cost. Their extensive application in medical imaging tasks, including cancer detection, cell classification, and tissue segmentation, further supports their suitability for analyzing complex microstructural patterns in electrospun nanofibrous materials.

### 3.3. Data augmentation and preprocessing

The performance of deep learning models critically depends on the size, diversity, and quality of the training dataset. In biomedical image classification tasks, such as distinguishing blood compatible and non-compatible electrospun nanofibre scaffolds, dataset sizes are often limited due to experimental constraints, high fabrication costs, and the requirement for expert annotation. Small datasets increase the risk of overfitting and reduce the generalizability of models. To address these limitations, data augmentation was employed to synthetically expand the dataset and introduce controlled variability, thereby improving model robustness.

Conventional data augmentation methods typically include geometric and photometric transformations, such as rotation, horizontal and vertical flipping, scaling and cropping, translation along the X or Y axes, and brightness or contrast adjustments. While these operations provide partial benefits, they are often static, low in diversity, and insufficient to capture the subtle morphological and textural variations inherent in microscopic biomedical images. In scanning electron microscopy (SEM) images of electrospun nanofibres, small differences in fiber alignment, surface morphology, and local contrast can be critical for accurate classification.

To overcome these limitations, the Albumentations library was adopted. Albumentations is a high-performance image augmentation framework that supports a wide range of geometric, photometric, and pixel-level transformations, including elastic deformation, motion blur, contrast limited adaptive histogram equalization (CLAHE), and additive noise. These transformations are well suited for SEM nanofibre images, preserving and mimicking intrinsic textural and morphological features.

Albumentations enables composable and probabilistic pipelines, allowing multiple transformations to be applied randomly during each training epoch. This approach ensures that each image generates a unique variant in every epoch, increasing variability without manual intervention [29]. Its optimized C++ backend and multiprocessing support allow fast augmentation compared to conventional frameworks such as Keras or PyTorch. In this study, transformations were selected to simulate variability introduced during electrospinning and SEM imaging, including fiber stretching, uneven illumination, surface irregularities, and local distortions.

Specifically, augmentations applied included random rotation, horizontal and vertical flipping, brightness and contrast adjustment, elastic distortion, and additive Gaussian noise. These operations helped the CNN models learn blood compatibility features independent of orientation, contrast, and local perturbations. Fig 3 illustrates the Albumentations pipeline implemented, while Fig 4 presents representative examples of augmented SEM images. Table 3 summarizes the Albumentations transformations evaluated and highlights those applied in this study, whereas Table 4 provides the detailed augmentation parameters and application probabilities used to ensure reproducibility.

**Table 3:** Summary of Albumentations transformations used in this study, highlighting those applied to SEM images for dataset augmentation.

Albumentations Transform	Used
RandomResizedCrop	✓
HorizontalFlip	✓
VerticalFlip	✓
RandomRotate90	✓
ElasticTransform	✓
GridDistortion	✓
OpticalDistortion	✓
GaussNoise	✓
GaussianBlur	✓
MotionBlur	✓
RandomBrightnessContrast	✓
HueSaturationValue	✓
CLAHE	✓
RandomGamma	✓
Sharpen	✓
Rotate	✗
ShiftScaleRotate	✗
Transpose	✗
RandomCrop	✗
CenterCrop	✗
Resize	✗
MedianBlur	✗
GlassBlur	✗
Emboss	✗
Cutout/ CoarseDropout	✗

**Table 4:** Detailed Albumentations augmentation parameters and application probabilities used for SEM image dataset generation and model training.

Augmentation	Parameters	Probability
RandomResizedCrop	scale = 0.98–1.00, ratio = 0.99–1.01	1.0
HorizontalFlip	NA*	0.05
VerticalFlip	NA	0.05
RandomRotate90	NA	0.02
ElasticTransform	α = 1, σ = 1	0.05
GridDistortion	distort_limit = 0.02	0.05
OpticalDistortion	distort_limit = 0.02, shift_limit = 0.02	0.05
GaussNoise	variance = 0–1	0.05
RandomBrightnessContrast	±0.02	0.05
RandomGamma	95–105	0.05

* NA – Not applicable.

**Fig 3:**
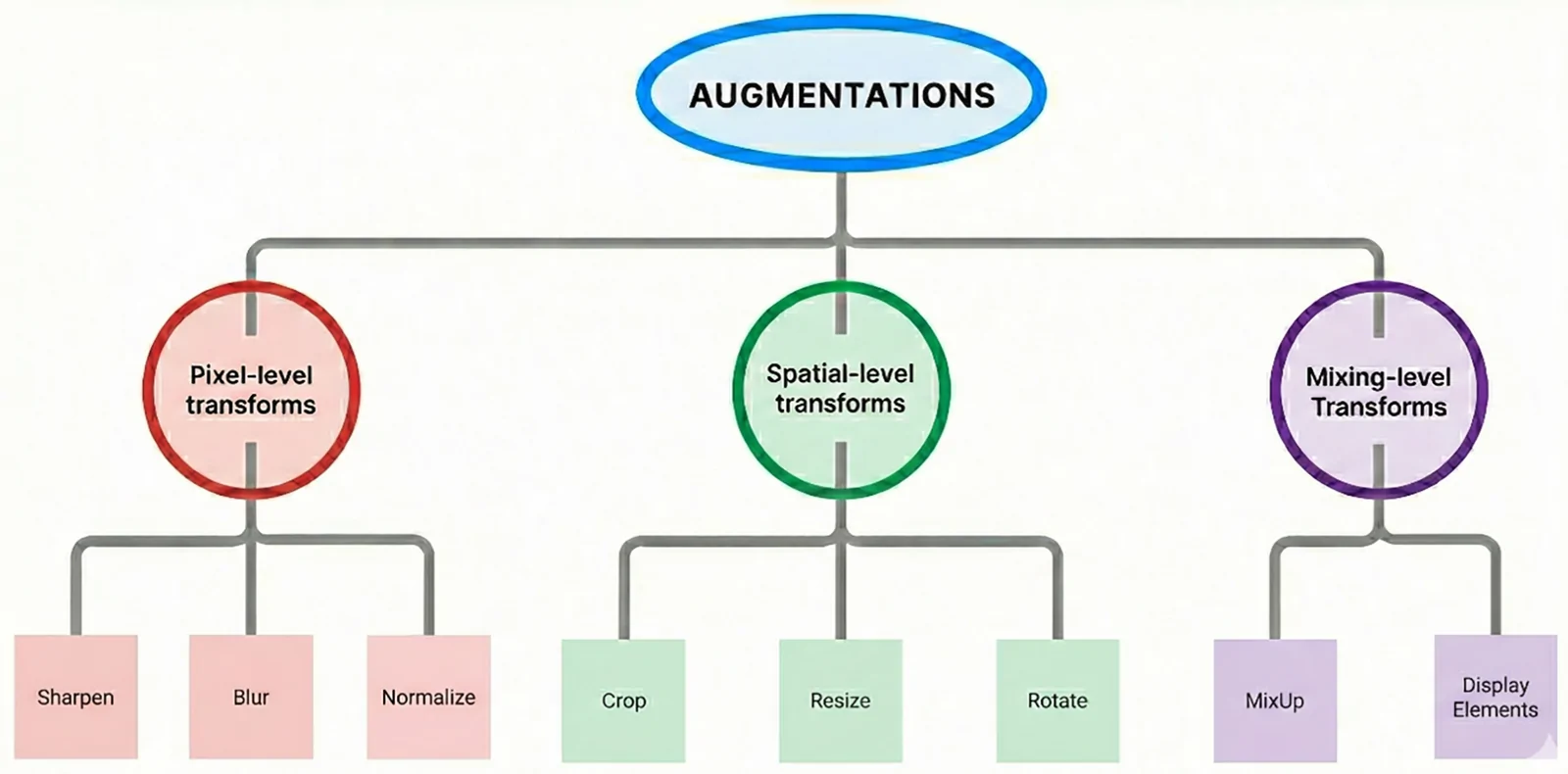
Schematic representation of the Albumentations-based augmentation pipeline, showing the sequence of transformations applied to nanofibre SEM images.

**Fig 4:**
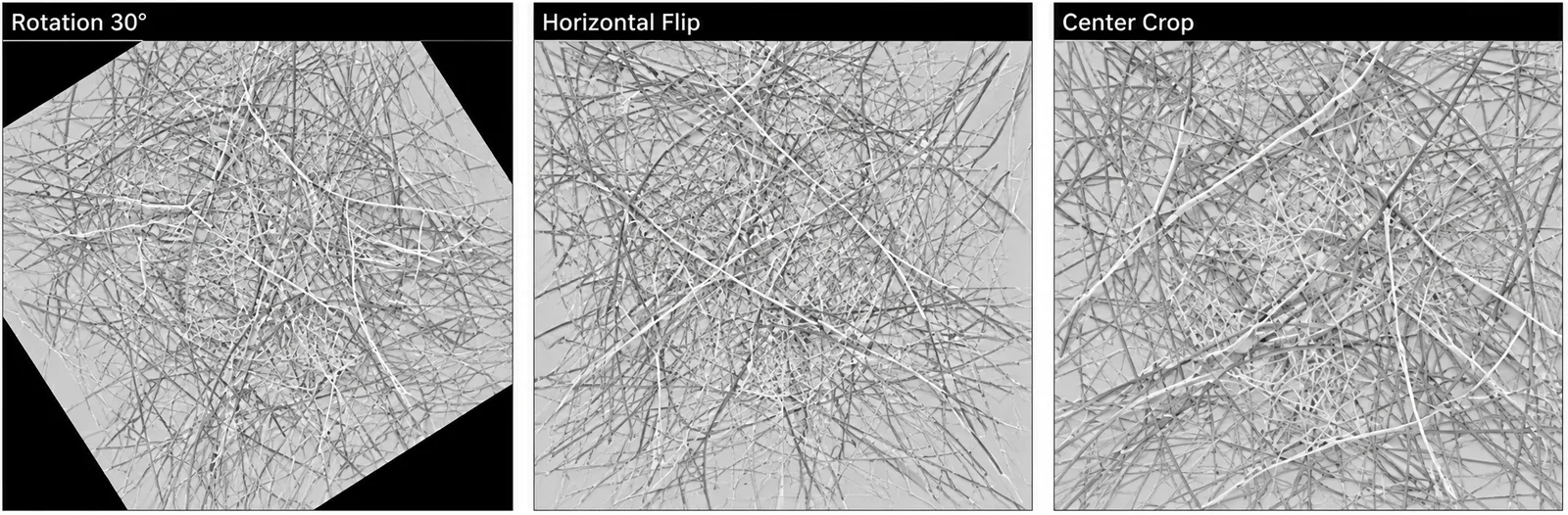
Representative examples of SEM images after applying Albumentations augmentations, demonstrating variation in fiber orientation, contrast, and texture.

The original SEM dataset consisted of labelled images of blood compatible and non-compatible nanofibre scaffolds, acquired at different magnifications. Table 5 presents the number of images in each class before and after augmentation. Following augmentation, the dataset was expanded to a total of 400 images, providing a balanced and diverse training set suitable for deep learning.

**Table 5:** Number of SEM images in each class (blood compatible and non-compatible) before and after augmentation at different magnifications.

Image Category	Number of images (Before Augmentation)		Number of images (After Augmentation)
Compatible	9	21	100
Compatible	12		100
Non-Compatible	15	17	100
Non-Compatible	2		100

Each CNN model is trained individually as a binary classifier to distinguish between the compatible and non-compatible fiber classes. The networks apply the binary cross-entropy loss function as given in equation 3.1


$$\begin{array}{c} L_{BCE}=-\;\frac{\text{1}}{N}\sum_{i=\text{1}}^{N}\left[y_{i}log(\hat{y_{i}})+(\text{1}-y_{i})log(\text{1}-\hat{y_{i}})\right] \end{array}$$ (3.1)

$$\begin{array}{c} where\;y_{i}\;\left\{\text{0},\text{1}\right\}\;is\;the\;true\;labe,\;\hat{y_{i}}\;[\text{0},\text{1}]\;is\;the\;predicted\;probability \end{array}$$

The binary cross-entropy loss function is well-suited for two-class problems, as it penalizes incorrect predictions proportionally to their confidence. Model optimization was performed using the Adam optimizer, which adaptively adjusts the learning rate for each parameter by combining the advantages of momentum and RMSProp, promoting stable convergence across various deep learning tasks. An initial learning rate of 1 × 10^−4^ was employed, and learning rate scheduling was applied to gradually reduce the rate during training to enhance convergence.

Training was conducted on a dataset of 400 SEM images after augmentation, evenly divided between compatible (200) and non-compatible (200) nanofibre samples. Augmentation was performed using the Albumentations library. Keras’ ImageDataGenerator was used to implement real-time data augmentation and normalization. A 75:25 split was applied to create training (300 images) and validation (100 images) sets. Each image was resized to 224 × 224 × 3 and fed to the network in mini-batches of 4, ensuring efficient and stable model training.

The fully connected top layers of the pre-trained convolutional neural networks (CNNs) VGG19, ResNet50, and InceptionV3 were replaced with a custom classification head tailored for binary classification of blood compatible and non-compatible nanofibres. The classification head consists of a Global Average Pooling (GAP) layer, which converts multi-dimensional feature maps into a single vector per channel, effectively reducing the number of parameters and mitigating overfitting. This is followed by a fully connected (Dense) layer with 256 neurons and Rectified Linear Unit (ReLU) activation, enabling non-linear feature learning from the extracted embeddings. A Dropout layer with a rate of 0.6 was applied to randomly deactivate 60% of neurons during training, further enhancing generalization and reducing overfitting. The final layer is a Dense output neuron with sigmoid activation, producing a probability score for binary classification between compatible (comp) and non-compatible (noncomp) nanofibres. Model performance was monitored on a separate validation set after each epoch, with the best-performing weights automatically saved for final evaluation.

After training, each CNN outputs a probability score representing the likelihood that a given input image belongs to the blood compatible class. For ensemble prediction, outputs from VGG19, ResNet50, and InceptionV3 were combined using a majority voting system. Each model’s probability was thresholded at 0.5 to generate a binary vote (equation 3.2), and the final class label was determined based on the majority rule, whereby at least two of the three models must agree on the class [30]. This straightforward approach enhances classification reliability by leveraging the complementary strengths of individual CNNs and mitigating errors due to model-specific biases.

The ensemble’s performance was rigorously evaluated on a separate test set not used during training or validation. Standard metrics, including accuracy, precision, recall, F1-score, and area under the receiver operating characteristic curve (ROC-AUC), were calculated. ROC-AUC quantifies the model’s ability to discriminate between compatible and non-compatible classes across all decision thresholds. By integrating predictions from multiple independent CNNs, the ensemble framework improves overall reliability, robustness, and generalization compared to single-model approaches. Conventional definitions of accuracy, precision, recall, specificity, and F1-score are provided in equation 3.3, serving as the basis for performance evaluation.


$$\begin{array}{c} \hat{y_{i}}=\;mode({\hat{y}}_{VGG\text{1}\text{9}},\;{\hat{y}}_{ResNet\text{5}\text{0}},\;{\hat{y}}_{InceptionV\text{3}}) \end{array}$$

$$\begin{array}{c} where\;each\;{\hat{y}}_{model}\;\in \left\{\text{0},\text{1}\right\}\;is\;the\;binary\;prediction\;model \end{array}$$ (3.2)

$$\begin{array}{cc} Accuracy\; & =\frac{TP+TN}{TP+TN+FP+FN},\;Precision\;=\frac{TP}{TP+FP},\;ecall\;\\ & =\frac{TP}{TP+FN}\;,\;Specificity\;=\frac{TN}{TN+FP},\text{ F1}-\text{score }=\text{2}\cdot \frac{Precision\times Recall}{Precision+Recall} \end{array}$$ (3.3)

To assess the reproducibility and stability of the proposed deep learning framework, each convolutional neural network (CNN) model was trained and evaluated through five independent runs using identical hyperparameter settings while allowing for random initialization and stochastic training variations. Performance metrics obtained from the five runs were subsequently analyzed using descriptive statistical measures, including the mean accuracy, standard deviation (SD), and relative standard deviation (RSD).

### 3.4. Explainable AI (XAI) for model interpretability

To enhance transparency and interpretability of the ensemble’s predictions, two explainable AI (XAI) methods were employed: Local Interpretable Model-Agnostic Explanations (LIME) and Integrated Gradients (IG).

LIME [24] generates local surrogate models to explain individual predictions. It perturbs the input image by modifying or masking superpixel regions and evaluates the resulting effect on the classifier’s output probability. This process identifies which regions of the image most strongly influence the model’s decision. The explanations are visualized as saliency maps or highlighted regions overlaid on the original image, allowing verification that the network focuses on meaningful structural features of the nanofibres.

Integrated Gradients [28] is a gradient-based attribution method that assigns an importance score to each pixel by integrating the gradients of the output prediction with respect to the input image along a linear path from a baseline (e.g., a blank image) to the actual input. This method satisfies desirable properties such as sensitivity and implementation invariance. The resulting heatmaps indicate pixels that contribute most strongly to the predicted class, providing pixel-level interpretability across the entire image.

In this study, both LIME and IG were applied to images from the compatible and non-compatible classes. LIME highlights influential local superpixel regions, while IG provides comprehensive pixel-level attribution. Together, these methods ensure that the CNNs attend to relevant fiber features rather than background artifacts, offering robust visual validation of model behavior. By combining a model-agnostic approach (LIME) with a gradient-based method (IG), the framework delivers clear, complementary insights into the ensemble’s decision-making process, fostering confidence in its reliability for biomedical image analysis and materials classification.

## 4. Results

This study employed deep learning classifiers to assess the blood compatibility of electrospun nanofibre materials. To ensure that data augmentation preserved the essential morphological features of the nanofibres, image similarity metrics were computed, including Mean Absolute Error (MAE), Mean Squared Error (MSE), and Peak Signal-to-Noise Ratio (PSNR). The results, summarized in Table 6, demonstrate that the augmented images maintain high fidelity with the original images for both compatible and non-compatible classes, confirming the reliability of the augmentation strategy for downstream deep learning analysis.

**Table 6:** Image similarity metrics (MAE, MSE, PSNR) for augmented SEM nanofibre images.

Class	MAE (norm)	MSE (norm)	PSNR (dB)
comp	0.2951	0.130275	57.06
noncomp	0.312	0.150439	56.39
overall	0.3021	0.138677	56.78

The Mean Absolute Error (MAE) quantifies the average absolute difference between the original and augmented image pixels, normalized to the range [0,1]. The Mean Squared Error (MSE) represents the average of squared pixel differences. Moderately low MAE and MSE values indicate that the augmentations were sufficiently strong to introduce variability while preserving essential morphological features, demonstrating effective augmentation. The Peak Signal-to-Noise Ratio (PSNR), expressed in decibels (dB), represents the ratio of signal strength to noise introduced during augmentation. PSNR values above 40 dB generally indicate that augmented images closely resemble the original. In this study, a PSNR of approximately 56 dB confirms that the augmentation process did not compromise image quality [31].

The experimental evaluation employed three state-of-the-art convolutional neural network (CNN) models — VGG19, ResNet50, and InceptionV3 — along with an ensemble model that combined their predictions. The dataset consisted of SEM images of electrospun nanofibres, classified as either blood compatible or non-compatible. The dataset was split into training and validation sets at a 75:25 ratio, resulting in 300 images for training and 100 images for validation. Model performance was assessed using validation accuracy, confusion matrices, precision, recall, F1-score, receiver operating characteristic (ROC) curves, precision-recall (PR) curves, and radar plots. Validation accuracy for all three models is shown in Fig 5. Among the individual CNNs, InceptionV3 achieved the highest classification performance, reaching a maximum accuracy of 99%. The ensemble model further enhanced stability and consistency across both classes, marginally outperforming InceptionV3 alone, highlighting the advantage of combining complementary CNN architectures for robust blood compatibility prediction of electrospun nanofibres. The reproducibility analysis demonstrated low variability across repeated experiments, with all models exhibiting RSD values below 3%, confirming the reliability and consistency of the proposed classification framework. The ensemble model achieved the lowest variability (RSD = 0.56%), indicating superior stability compared with the individual CNN architectures (Table 7).

**Table 7:** Reproducibility assessment of CNN models using mean, SD, and RSD across five independent runs.

Model	Run1	Run2	Run3	Run4	Run5	Mean	Std. Dev.	RSD% $\frac{Std.Dev}{Mean}*100$
VGG19	98	98	96	98	96	97.2	1.095	1.13%
Resnet50	80	80	84	82	82	81.6	1.693	2.05%
InceptionV3	99	99	100	98	99	99.0	0.707	0.71%
Ensemble	99	99	98	98	98	98.4	0.548	0.56%

**Fig 5:**
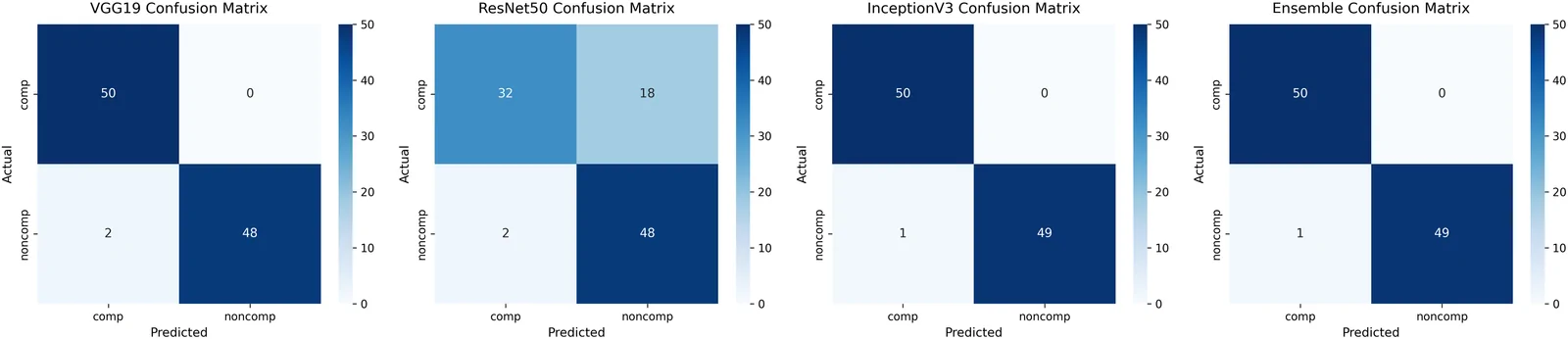
Confusion matrices for individual CNN models (VGG19, ResNet50, InceptionV3) and the ensemble model, showing the distribution of true positives, true negatives, false positives, and false negatives in the classification of compatible and non-compatible electrospun nanofibre SEM images.

Confusion matrices (Fig 5) were computed for each model to evaluate classification performance between compatible and non-compatible nanofibre classes. These matrices illustrate the distribution of true positives, true negatives, false positives, and false negatives, providing a detailed overview of model accuracy and misclassification patterns. Precision, recall, and F1-scores for all models are summarized in Table 8. Both InceptionV3 and the ensemble model consistently outperformed the other architectures, achieving near-perfect balance across all metrics.

**Table 8:** Performance metrics (Precision, Recall, F1-score) of individual CNN models and the ensemble model for classification of compatible and non-compatible electrospun nanofibre SEM images.

Model	Recall	Precision	F1-Score
VGG19	0.962	1.0	0.981
ResNet50	0.941	0.640	0.763
InceptionV3	0.98	1.0	0.99
Ensemble	0.98	1.0	0.99

The area under the receiver operating characteristic curve (AUC-ROC) was used to assess each model’s ability to discriminate between compatible and non-compatible classes across different decision thresholds. An ROC curve closer to the top-left corner indicates high true positive rates (sensitivity) with low false positive rates (1-specificity), whereas a curve along the diagonal reflects random performance. AUC values range from 0 to 1, with 0.5 representing random guessing and values closer to 1 indicating superior discrimination ability. Specifically, AUC values of 0.9–1.0 indicate excellent performance, 0.8–0.9 good, 0.7–0.8 fair, 0.6–0.7 poor, and below 0.5 worse than random [32].

Fig 6 presents the ROC curves for all models. Both InceptionV3 and the ensemble model closely follow the ideal curve, achieving an AUC of 1.000, demonstrating excellent discrimination between compatible and non-compatible classes. VGG19 and ResNet50 achieved AUC values of 0.999 and 0.979, respectively. Precision-recall (PR) curves further confirm the superior performance of InceptionV3 and the ensemble model, showing high precision even as recall varies, indicating low false positive rates while maintaining effective sensitivity. These results collectively highlight the robustness and reliability of the ensemble approach in predicting blood compatibility of electrospun nanofibres.

**Fig 6:**
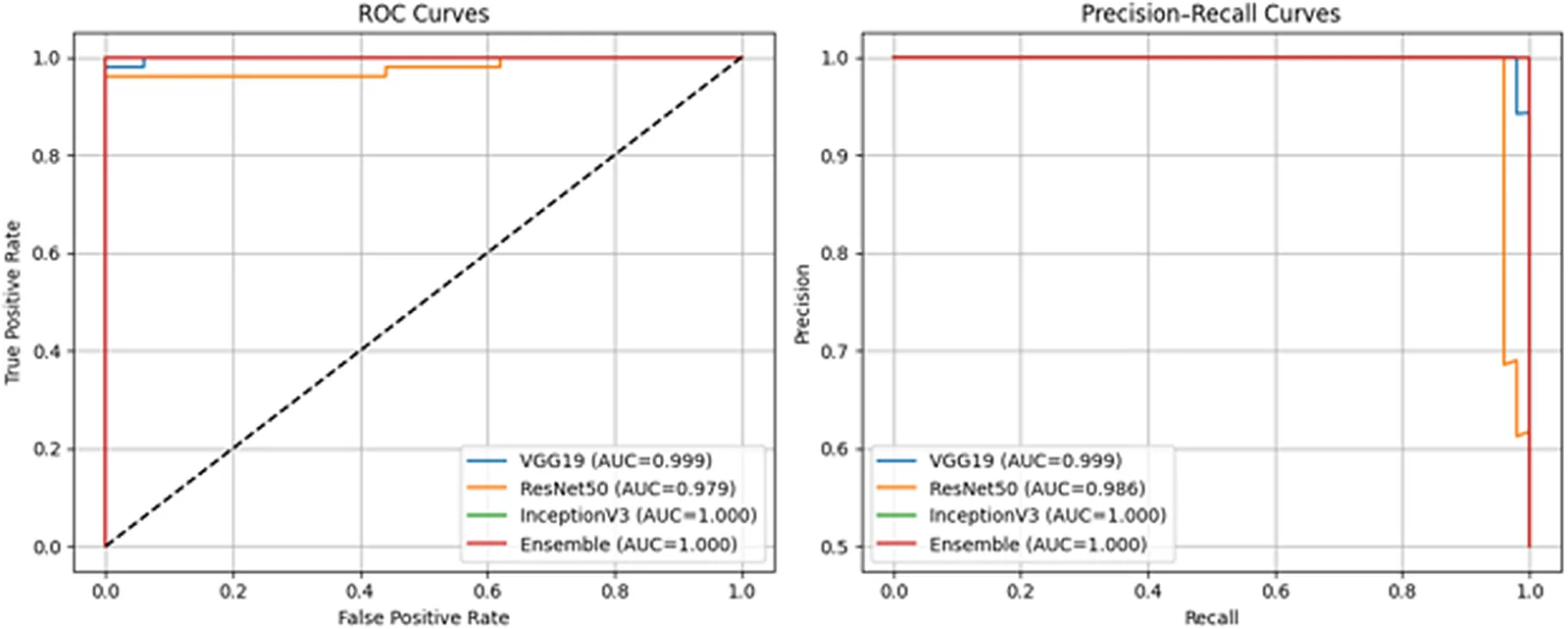
Receiver Operating Characteristic (ROC) curves for VGG19, ResNet50, InceptionV3, and the ensemble model, illustrating each model’s ability to discriminate between compatible and non-compatible nanofibre classes and the Precision-Recall (PR) curves for the same models, highlighting precision and recall trade-offs and confirming the ensemble and InceptionV3 models’ superior performance.

The learning behavior of VGG19, ResNet50, and InceptionV3 was evaluated by plotting training and validation loss, as well as training and validation accuracy, across epochs. VGG19 exhibited fluctuations in validation loss during the early epochs, reflecting sensitivity to weight initialization. Although it stabilized after approximately 10 epochs, slight underfitting was observed compared to the deeper models. ResNet50 showed smoother convergence, with steadily decreasing validation loss and increasing accuracy; the residual connections facilitated efficient gradient propagation and improved training stability. InceptionV3 demonstrated the most stable training behavior, with rapid reduction in loss and consistently high validation accuracy, achieving near-perfect classification within the first few epochs. Figs 7–9 present the training and validation curves for all models, confirming that

**Fig 7:**
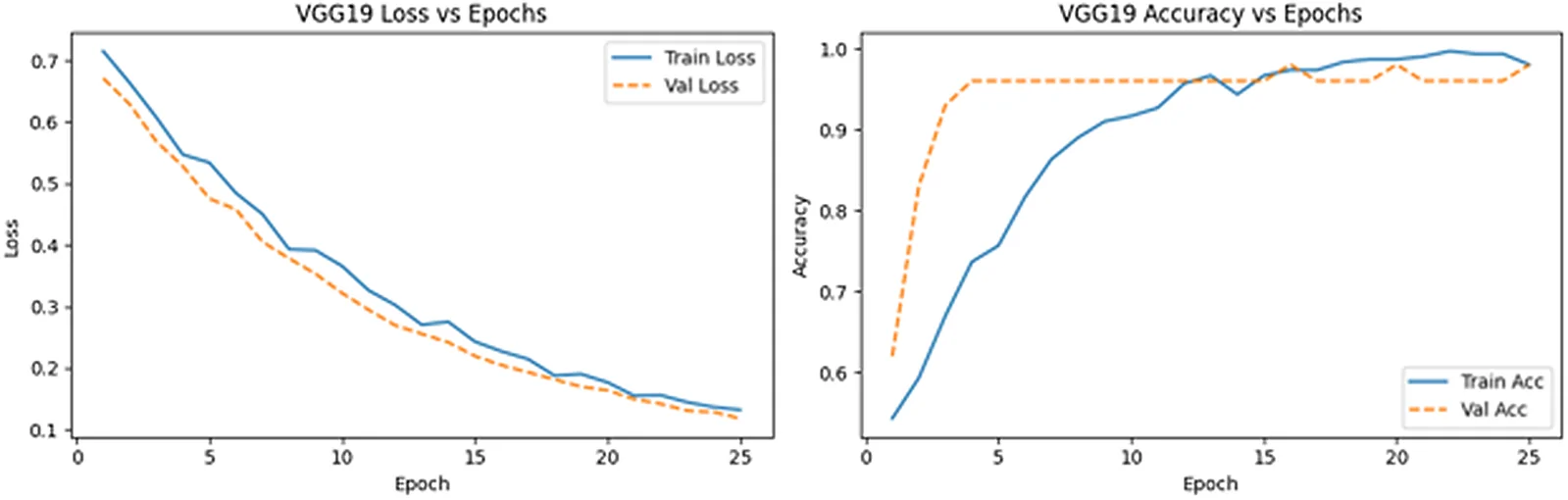
Training and validation loss versus epochs and accuracy versus epochs of VGG19 for classification of compatible and non-compatible electrospun nanofibre SEM images over 25 epochs.

**Fig 8:**
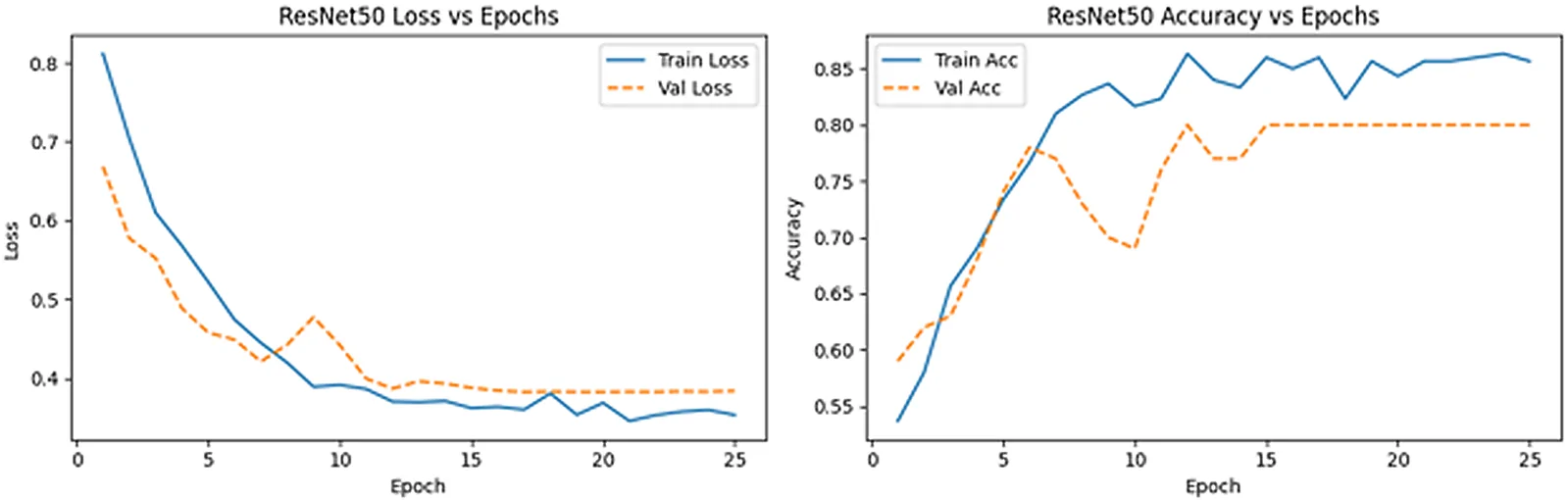
Training and validation loss versus epochs and accuracy versus epochs of ResNet50 for classification of compatible and non-compatible electrospun nanofibre SEM images over 25 epochs.

**Fig 9:**
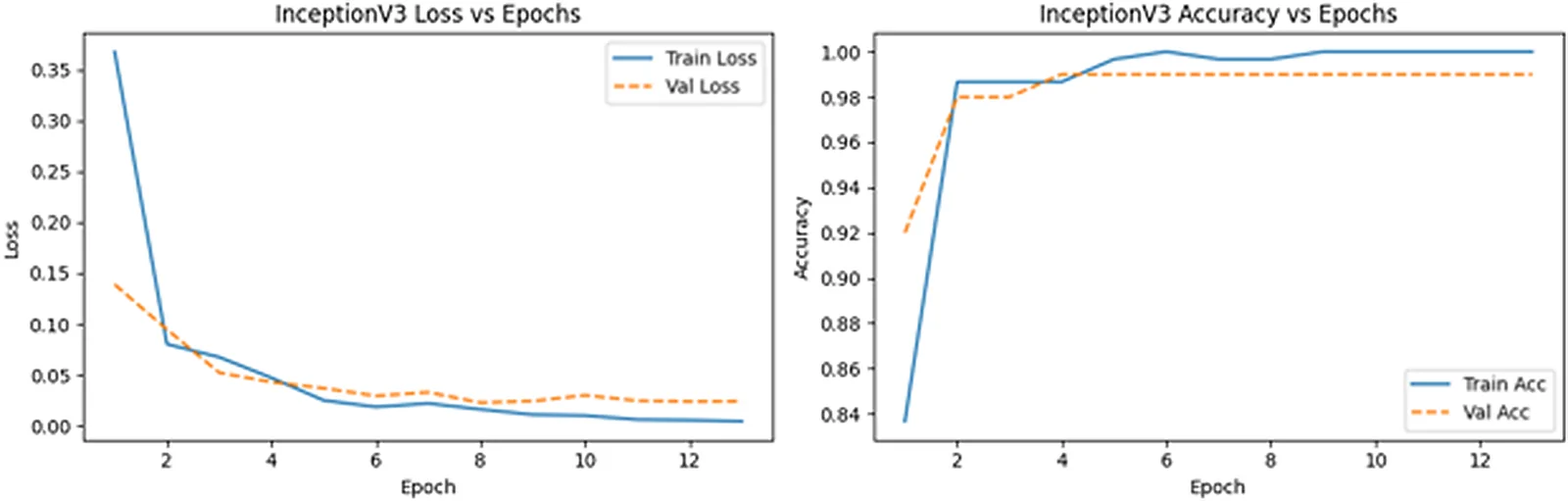
Training and validation loss versus epochs and accuracy versus epochs of Inception V3 for classification of compatible and non-compatible electrospun nanofibre SEM images over 25 epochs.

InceptionV3 generalizes best, with minimal divergence between training and validation curves. These results highlight the efficiency and robustness of the InceptionV3 architecture in capturing the morphological features necessary for accurate classification of compatible and non-compatible electrospun nanofibres.

Fig 10 presents a radar plot comparing the performance of VGG19, ResNet50, and InceptionV3 models across four evaluation metrics: accuracy, specificity, Matthews Correlation Coefficient (MCC), and Cohen’s Kappa. Accuracy reflects the overall correctness of the model’s predictions. Specificity quantifies the model’s ability to correctly identify negative samples (here, class “non-comp”), indicating robustness against false positives. MCC, ranging from −1 to +1, accounts for all elements of the confusion matrix, with +1 representing perfect prediction and −1 indicating complete disagreement. Cohen’s Kappa measures agreement between predicted and actual labels, adjusted for chance, where values closer to 1 indicate stronger concordance. In the radar plot, a model’s polygon approaching the outer edge (value 1.0) signifies superior performance across all metrics. InceptionV3, VGG19, and the Ensemble model exhibited substantially higher performance than ResNet50, with their polygons nearly overlapping the optimal values for each metric. In contrast, ResNet50 showed lower scores, particularly in MCC, Kappa, and specificity, highlighting its relative underperformance in distinguishing between compatible and non-compatible cases.

**Fig 10:**
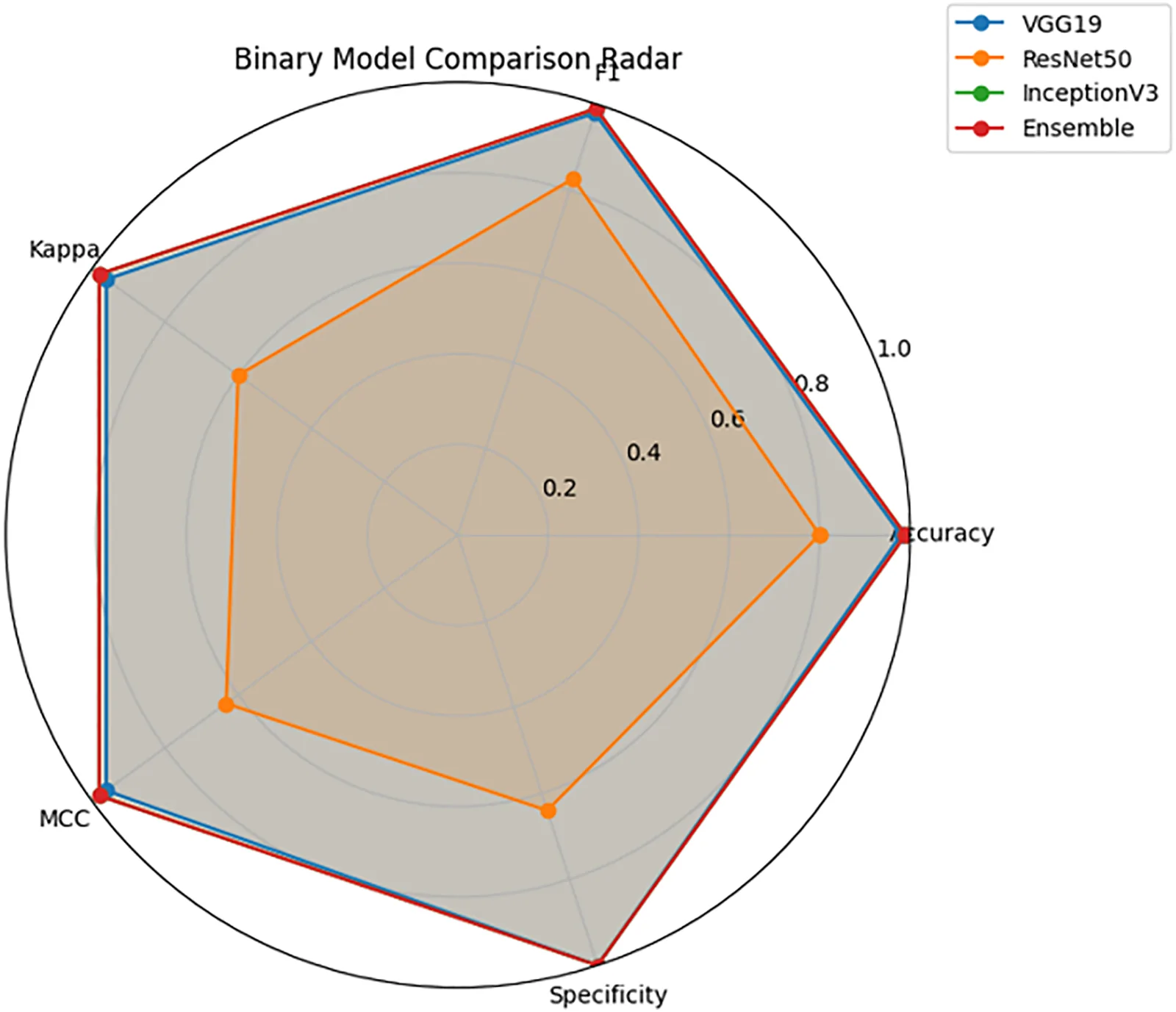
Radar plot comparing the performance of VGG19, ResNet50, InceptionV3, and the Ensemble model across accuracy, specificity, Matthews Correlation Coefficient (MCC), and Cohen’s Kappa. Higher values indicate better performance across all metrics, with InceptionV3, VGG19, and the Ensemble outperforming ResNet50.

### 4.1. Explainability (XAI)

To enhance the interpretability of the proposed ensemble framework, we applied Local Interpretable Model-Agnostic Explanations (LIME) and Integrated Gradients (IG) to the predictions generated by each backbone model: VGG19, ResNet50, and InceptionV3. For each model, two images are presented: one showing LIME-based superpixel segmentation and the other displaying the IG-based attribution map. LIME highlights localized superpixel regions that substantially influenced the model’s decision, allowing visual verification that the CNN focused on relevant fiber structures rather than background noise. In contrast, IG produces pixel-level attribution heatmaps, capturing fine-grained gradient contributions from the input image. Together, LIME and IG provide complementary insights into the model’s decision-making process. Figs 11–16 illustrates the LIME and IG results for all three backbone models.

**Fig 11:**
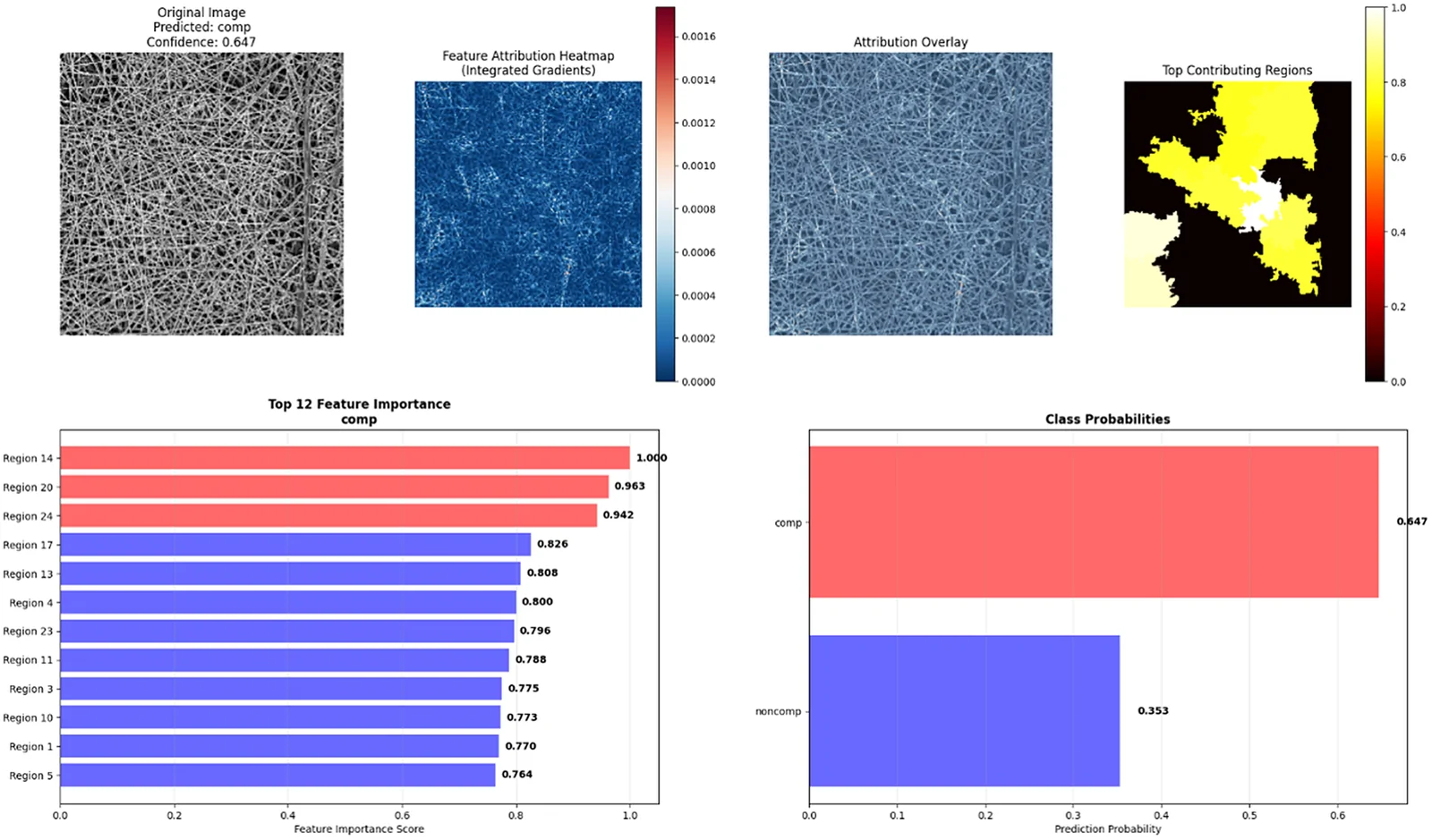
Integrated Gradients (IG) attribution map for VGG19, highlighting fine-grained pixel contributions along fiber boundaries. Maximum attribution: 0.001735, mean attribution: 0.000121, top region importance: 1.

**Fig 12:**
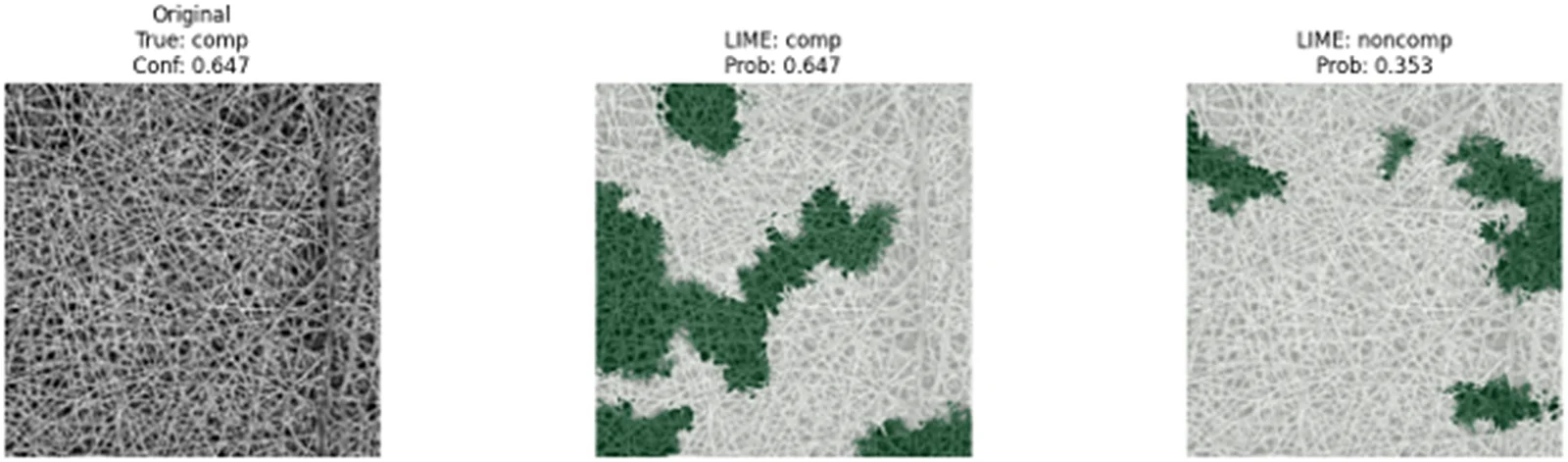
LIME-based explanation for VGG19, highlighting the superpixel regions most influential in the model’s classification.

**Fig 13:**
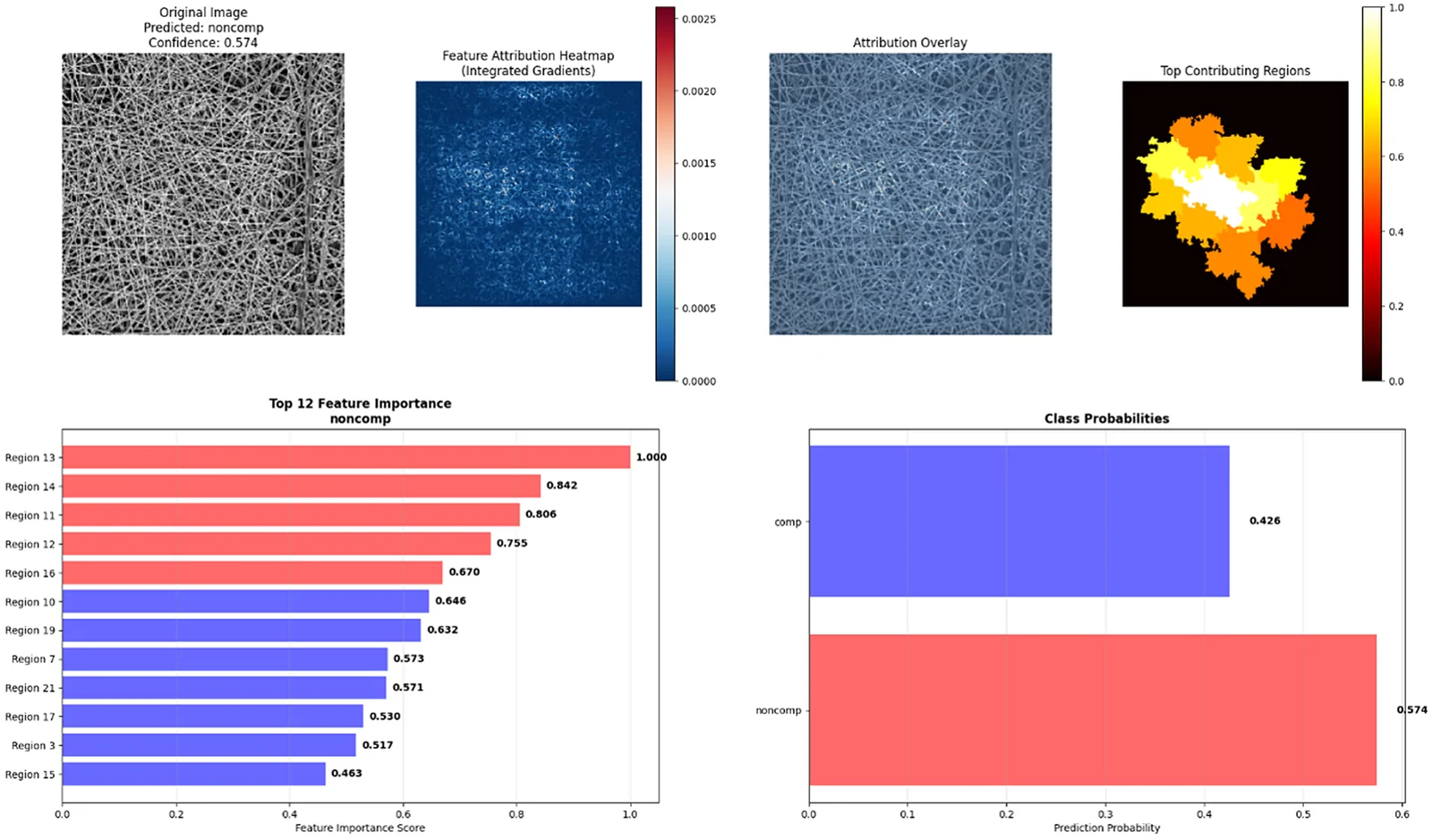
Integrated Gradients (IG) attribution map for ResNet50, highlighting fine-grained pixel contributions along fiber boundaries. Maximum attribution: 0.002577, mean attribution: 0.000100, top region importance: 1.

**Fig 14:**
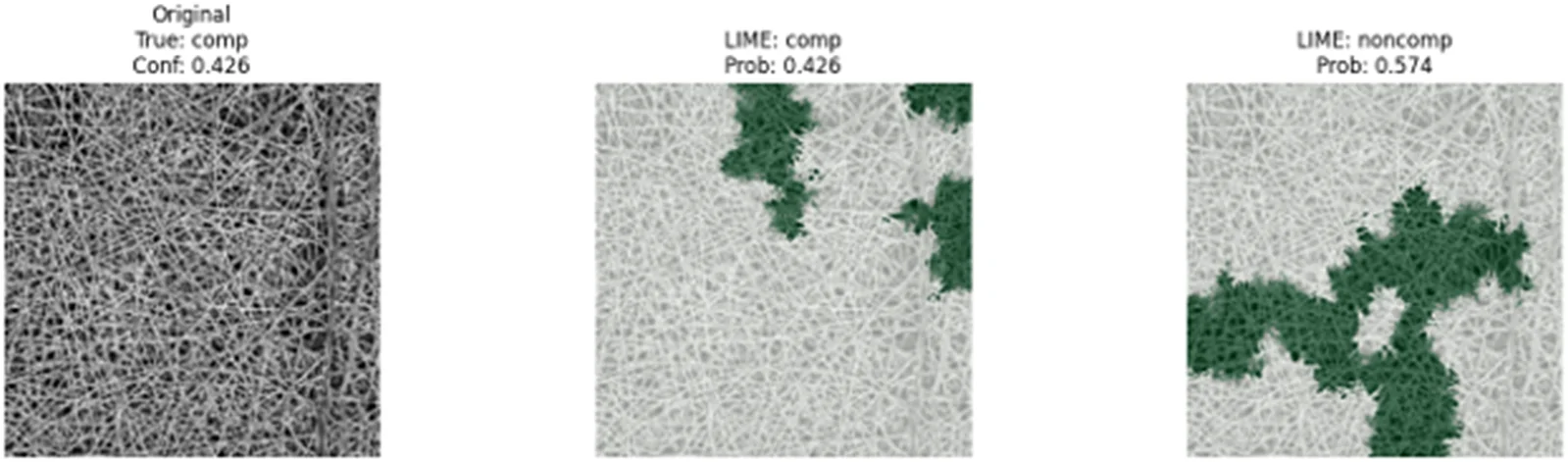
LIME-based explanation for ResNet50, highlighting the superpixel regions most influential in the model’s classification.

**Fig 15:**
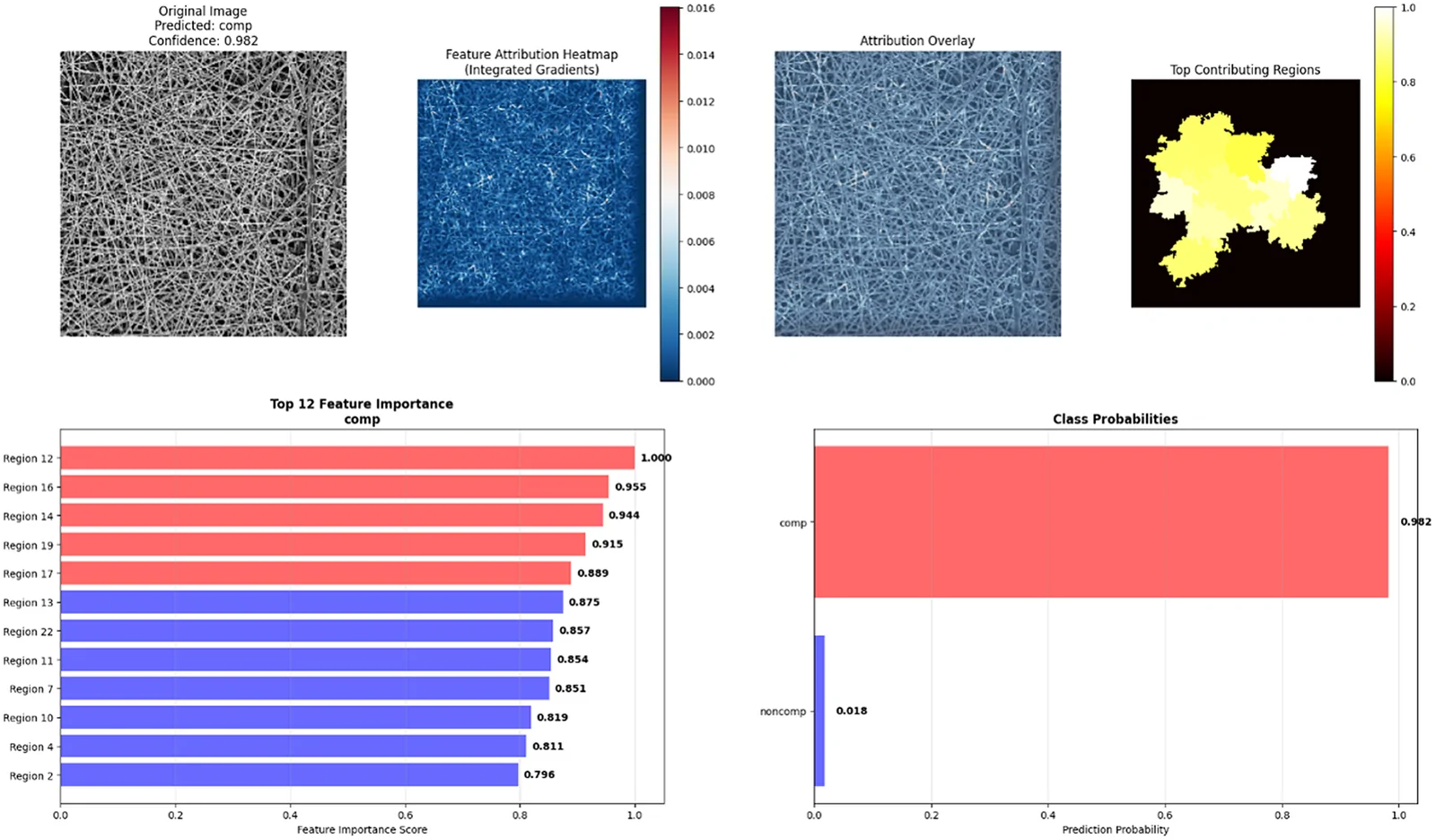
Integrated Gradients (IG) attribution map for InceptionV3, highlighting fine-grained pixel contributions along fiber boundaries. Maximum attribution: 0.016045, mean attribution: 0.001239, top region importance: 1.

**Fig 16:**
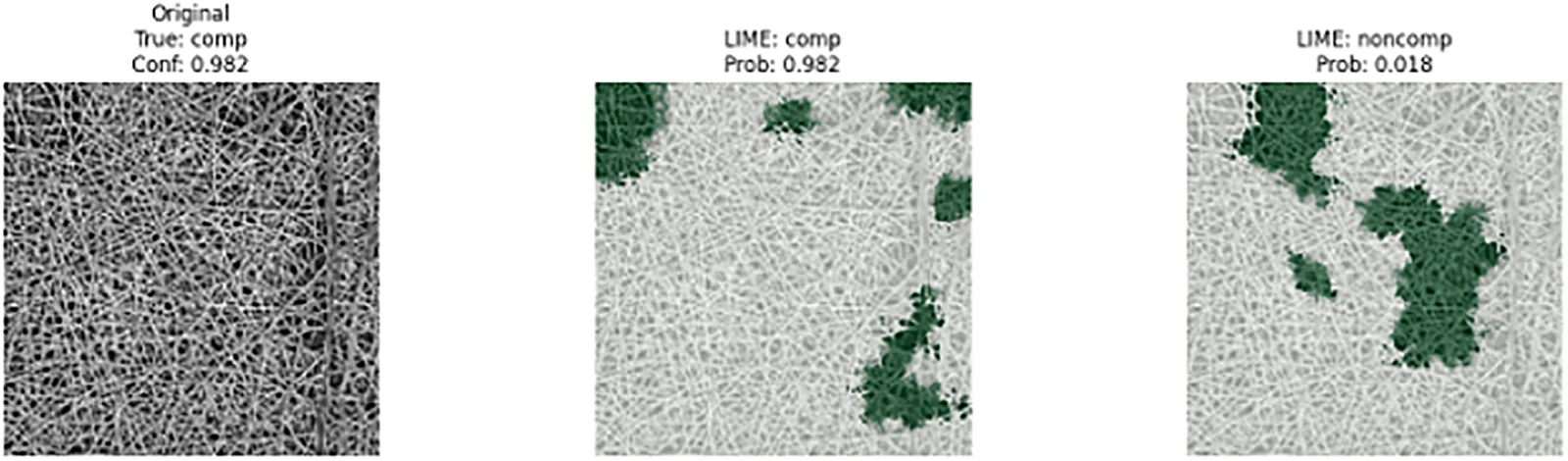
LIME-based explanation for InceptionV3, highlighting multi-scale super pixel regions across the fiber mat that most influenced the model’s classification.

## 5. Discussion

The fidelity analysis of data augmentation further validates the reliability and reproducibility of the proposed framework. The consistently low mean absolute error (MAE) and mean squared error (MSE) values, together with the high peak signal-to-noise ratio (PSNR), confirm that the augmentation strategy preserved the intrinsic morphological structure of electrospun nanofibres without introducing distortions that could confound feature learning. This validation is particularly important for microscopy-based image datasets, where excessive or poorly controlled augmentation may cause deep learning models to learn artificial or non-physical patterns rather than genuine microstructural characteristics. For electrospun nanofibres, the combination of geometric Flip/Rotate and Scale/Zoom transformations represents a highly effective augmentation strategy, as these operations preserve key morphological descriptors such as fibre diameter and porosity, which are critical parameters in biomaterials research [33].

The results demonstrate that deep learning architectures can reliably differentiate between blood-compatible and non-compatible electrospun nanofibre materials using only morphological information derived from SEM images, without incorporating direct blood–material interaction data. High performance across multiple magnification levels indicates that compatibility-associated microstructural features are consistently preserved across imaging scales, enabling convolutional neural networks to learn and generalise scale-invariant morphological representations. The ensemble model further enhanced robustness, delivering consistently high F1-score, specificity, Matthews Correlation Coefficient (MCC), and Cohen’s Kappa values, comparable to or exceeding those of the best-performing individual architecture.

Integrating complementary feature representations from VGG19, ResNet50, and InceptionV3 under an ensemble framework proved advantageous, particularly in biomedical imaging datasets where subtle morphology-driven differences dictate classification outcomes. Majority-voting aggregation mitigates model-specific biases, reduces variance, and improves generalisation reliability, which is essential for practical deployment as a sustainable pre-screening tool. The superior performance of the ensemble model further highlights the value of architectural diversity in capturing complementary microstructural information from SEM images.

Key morphological variables, including fibre diameter, pore size, fibre density, and surface uniformity, are known to influence protein adsorption, platelet activation, and thrombus formation, indicating that SEM-based physicochemical analysis can serve as a valuable pre-screening tool for hemocompatibility assessment. By leveraging imaging-derived microstructural features, this approach reduces dependence on biological testing, blood samples, and laboratory consumables during the early stages of biomaterial development. Importantly, the framework aligns with ISO 10993 guidelines, where physicochemical characterisation is recognised as a foundational step for planning subsequent biological evaluation.

Explainable Artificial Intelligence (XAI) techniques further elucidated mechanistic relationships between nanofibre morphology and blood compatibility, addressing concerns regarding the “black-box” nature of deep learning models. LIME and Integrated Gradients consistently identified nanofibre density, orientation, and structural homogeneity as the most influential factors driving classification decisions. These morphological characteristics correspond closely with established hemocompatibility mechanisms, including platelet activation, protein adsorption behaviour, and thrombus formation. Uniform fibre distribution and surface homogeneity may suppress non-specific protein adsorption, whereas regulated porosity and fibre density influence local flow behaviour and stagnation regions that contribute to thrombogenic responses. The strong agreement between XAI-derived feature attributions and established hemocompatibility principles suggests that the proposed framework captures meaningful structure–function relationships within large SEM datasets, providing both predictive accuracy and mechanistic interpretability.

Haemocompatibility is a multifactorial property governed not only by surface morphology but also by surface chemistry, hydrophobicity, surface energy, charge distribution, protein adsorption behaviour, and flow-dependent biological responses that are not directly captured by SEM images. Consequently, the proposed framework should be regarded as a rapid, morphology-driven pre-screening tool rather than a complete physicochemical predictor of blood compatibility. Within the controlled electrospun systems investigated, morphological features extracted from SEM images are indirectly influenced by material composition and fabrication parameters, enabling deep learning models to learn meaningful structure–function relationships when trained using experimentally validated haemocompatibility labels. While the framework is not intended to replace mandatory ISO 10993-compliant biological evaluation, it can substantially reduce experimental burden by prioritising promising candidates for subsequent testing. Predictive performance may be affected when substantial changes in surface chemistry occur without corresponding morphological variations, highlighting the need for future multimodal approaches integrating morphology, surface chemistry, wettability, and compositional descriptors.

The proposed framework is intended as a rapid and supportive pre-screening tool rather than a replacement for conventional biological assays. The model demonstrated strong predictive performance across multiple evaluation metrics, including accuracy, precision, recall, and F1-score, while Explainable Artificial Intelligence (XAI) techniques enhanced interpretability by highlighting SEM image regions that most strongly influenced classification outcomes. Importantly, the framework utilises SEM-derived morphological and physicochemical features associated with surface–blood interactions, enabling scientifically relevant hemocompatibility prediction. However, its predictive performance remains dependent on dataset diversity, imaging quality, and model generalisability. Despite these limitations, the impact of the present study lies in demonstrating that SEM-derived morphological information contains meaningful predictive signals associated with hemocompatibility. Although developed using polyurethane-based electrospun nanofibres, the framework establishes a proof-of-concept for applying explainable artificial intelligence to extract biologically relevant information from routinely acquired SEM micrographs. Following appropriate training and validation, the methodology may be extended to other electrospun biomaterial systems. The impact of the proposed framework can be summarised in three key aspects: (i) reducing experimental burden, resource consumption, and reliance on blood samples during early-stage biomaterial screening; (ii) supporting more efficient biomaterial development and candidate prioritisation prior to comprehensive biological evaluation; and (iii) promoting the integration of explainable artificial intelligence into biomaterials characterisation while aligning with the principles of the 3Rs (Replacement, Reduction, and Refinement).

Therefore, the proposed approach should be interpreted as a complementary decision-support system for early-stage biomaterial screening rather than a standalone predictive replacement for experimental testing. By enabling rapid identification of promising candidates, the framework can reduce experimental burden, resource consumption, and laboratory waste. Furthermore, the approach aligns with the principles of the 3Rs (Replacement, Reduction, and Refinement), which are increasingly emphasised within UK research and regulatory frameworks to minimise reliance on animal experimentation where scientifically appropriate. Nevertheless, final validation using established biological assays remains essential for clinical translation and regulatory approval.

Taken together, these results demonstrate that interpretable deep learning enables accurate classification while linking model predictions to physically meaningful morphological features. This dual capability supports reproducibility, data-driven evaluation, and sustainability advantages in biomaterials research. By reducing dependence on blood-based assays and laboratory consumables during early-stage screening, the framework has the potential to accelerate material development while supporting responsible and regulatory-compliant biomedical device innovation. Although the current study focuses on binary classification, the insights provided by XAI methods offer opportunities for the rational design and optimisation of nanofibre architectures with enhanced hemocompatibility, thereby supporting sustainable innovation in medical devices and broader global health technologies [34].

Nonetheless, several limitations should be acknowledged. Despite spanning multiple magnification levels and a range of scaffold morphologies, the dataset may not fully capture variability arising from different electrospinning parameters, polymer compositions, surface chemistries, or physiological blood-flow conditions. In addition, the framework relies exclusively on SEM-derived morphological information and therefore does not directly account for dynamic blood–material interactions such as time-dependent protein adsorption, platelet activation kinetics, complement activation, or coagulation cascade progression, which are typically evaluated through biochemical and flow-based assays [35]. Future work should focus on expanding dataset diversity, incorporating multimodal characterisation techniques including surface chemistry analysis, wettability measurements, atomic force microscopy, and standardised hemocompatibility assays and exploring generative or inverse-design approaches to establish more comprehensive relationships between material properties and blood compatibility outcomes.

Table 9 compares the proposed framework with existing state-of-the-art methods, highlighting its ability to deliver automated, quantitative, and interpretable analyses while overcoming limitations associated with manual, labour-intensive, or “black-box” approaches.

**Table 9:** Comparison of the proposed method with state-of-the-art approaches in the literature.

References	Material/focus	Methodology	Analysis Type	Key Limitation
[35] Chen, S., et al (2017)	Comprehensive review of electrospun nanofibres tailored for wound care and biological matrix healing.	Review and meta-analysis of standard electrospinning fabrication parameters and biological functionalization techniques.	Qualitative/ Structural review of cellular interactions and matrix design.	Lacks standard scaling parameters for industrial manufacturing; transition from lab-scale setups to clinical translation remains challenging.
[36] Anugya Bhatt et al[2022]	Evaluation of blood-contacting medical products and general biomedical material safety profiles.	Comprehensive in vitro blood compatibility assessment protocols (hemolysis assays, coagulation times, platelet activation).	Experimental Screening/ Regulatory compliance and physiological safety evaluation.	High statistical variability in manual assay screenings; lacks automated mathematical or algorithmic standardization for complex topography.
[37] Anjum, S., et al. (2023)	PVP/PVA biomimetic electrospun nanofibrous tissue scaffolds.	Optimization via Design of Experiments (DoE) (Central Composite Design matrix), combined with SEM imaging and in vitro hemolysis/cytotoxicity profiles.	Statistical Optimization & Experimental Verification.	DoE models handle global processing boundaries well but do not provide real-time feature segmentation of complex microscopic structures.
[38] Clauser, J. C et al. [2021]	Polyurethane and diverse blood-contacting biomaterial surfaces.	Automated image processing using custom machine learning segmentation algorithms to evaluate platelet adhesion over vast surface areas.	Automated Computational Computer Vision/ Image Segmentation.	The trained algorithm relies heavily on specific microscopy contrast inputs and requires highly uniform image quality for precise feature extraction.
[39] Subeshan, B et al. (2024)	Comprehensive state-of-the-art review on Machine Learning (ML) applied to electrospun nanofibres.	Critical analysis of ML algorithms (CNNs, Random Forests, SVMs) used to predict fiber diameter, web morphology, and structural performance.	Algorithmic Review/ Data-Driven Modeling.	Highly dependent on the availability of large, curated, and standardized open-source microscopic datasets for model training.
Proposed Method (2025)	Blood Compatible/Non -Blood – Compatible Nanofibres	Albumentations based augmentation+ DL + XAI	Interpretable Quantitative	–

Overall, this study establishes a robust and scientifically grounded foundation for the application of explainable deep learning in blood compatibility assessment. The proposed framework demonstrates high predictive performance, mechanistic interpretability, and strong potential for accelerating preclinical screening while supporting the rational design of electrospun nanofibre systems with enhanced clinical applicability.

## 6. Conclusion

This study demonstrates a high-precision and interpretable framework for automated analysis of electrospun nanofibres using deep learning combined with Explainable Artificial Intelligence (XAI). By leveraging SEM images acquired at multiple magnifications and employing Albumentations-based data augmentation to expand the dataset to 400 images, the framework addresses the common challenge of limited data availability in biomaterials research while preserving microstructural fidelity. Individual models such as VGG19 and ResNet50 provided strong baseline performance; however, the InceptionV3 architecture integrated within an ensemble voting framework achieved the highest predictive performance, reaching a peak accuracy of 99%.

Importantly, the integration of XAI techniques, including Integrated Gradients and LIME, enabled mechanistic interpretability by demonstrating that model predictions were driven by meaningful nanofibre morphological characteristics rather than imaging artefacts. This enhances transparency, reproducibility, and confidence in the classification process while addressing limitations commonly associated with conventional “black-box” deep learning models.

Beyond predictive performance, the proposed framework offers important sustainability and translational advantages. By enabling rapid morphology-based pre-screening of biomaterials using routinely acquired SEM images, the approach can reduce dependence on extensive biological testing, blood samples, and laboratory consumables during the early stages of material development. In doing so, it supports more efficient candidate prioritisation, minimises experimental waste, and aligns with ISO 10993 principles that recognise physicochemical characterisation as an important component in planning biological evaluation.

Although the framework is not intended to replace established hemocompatibility assays or regulatory biological testing, it provides a complementary decision-support tool for early-stage biomaterial screening. Overall, this study establishes a scalable, reproducible, and interpretable strategy for AI-assisted evaluation of electrospun nanofibre systems, supporting accelerated biomaterial development, data-driven design optimisation, and responsible innovation in blood-contacting biomedical technologies.
